# Trend of estimated participation rate by regional block, gender, and age group in the 1997–2019: National Health and Nutrition Survey in Japan

**DOI:** 10.1371/journal.pone.0286169

**Published:** 2024-03-13

**Authors:** Midori Ishikawa, Osamu Hemmi, Hidemi Takimoto, Mai Matsumoto, Tetsuji Yokoyama

**Affiliations:** 1 Department of Health Promotion, National Institute of Public Health, Wako, Saitama, Japan; 2 Department of Nutritional Epidemiology and Shokuiku, National Institute of Health and Nutrition, National Institutes of Biomedical Innovation, Health and Nutrition, Settsu, Osaka, Japan; JABSOM: University of Hawai’i at Manoa John A Burns School of Medicine, UNITED STATES

## Abstract

This study aimed to identify the trend of approximate participation rate in Japan’s National Health and Nutrition Survey (NHNS_J). The proportion of participants among all residents of surveyed districts (estimated participation rate: EPR) was calculated by 12 regional blocks, gender, and age group, and the trend and annual percent change (APC) were clarified. Based on the 1997–2019 NHNS_J data, we created a database classified by prefecture, gender, and age group; in addition to these, the number of people per household by Population Census or population estimates were obtained from e-Stat and added to the database. All analyses were performed by regional block and gender and EPR for each year was calculated by the age group. Trends of EPR, overall and by the age group, were presented using graphs, illustrating the exponential regression curve. The graphs showed APC, standard error, and statistical significance by age group. The EPRs were declining in all the regional blocks. Additionally, the rates of decline in APC in young people under the age of 50 years were higher than those in the older age groups in 9 of 12 regional blocks. The nationwide APC in the age group <50 years was significantly larger than that in the age group ≥50 years. The declining EPR trend in NHNS_J in all regional blocks (especially among younger people) suggests the need for a strategy to improve participation rates in the future.

## Introduction

Japan’s National Health and Nutrition Survey (NHNS_J) was started in 1945 and has been conducted every year since; it has recorded the health and nutritional status and food and nutrient intake over the years [1]. The data have been used for framing and evaluating national health promotion policies, such as “Health Japan 21” [2, 3]. However, the participation rate has declined in recent years [4], particularly among younger age groups, males, and single-person households [5].

Low participation rates may have serious implications for the generalizability of survey results and the accuracy of any conclusions based on survey data [6–9]. The Ministry of Health, Labor, and Welfare in Japan has revised the survey protocol to increase the participation rate, yet it is gradually declining [10].

In addition, the situations of residents’ participation rate and the measures to promote participation in the survey were different in local governments [11].

Furthermore, the total population has decreased in recent years, and the number of people per household has been decreasing over several years in Japan [12]. The latter factors include decreases in large-family households, increases in single-person households, and changes in familial structure. People’s lifestyles have been diversifying, and these situations differ by region [12]. In other countries, it has been reported that the population structure affects the response rate of surveys [13, 14] and may lead to regional differences [15].

Thus, it is important to assess the long-term trends of the participation rate in the NHNS_J by region. The trends of participation rate of the whole country have been reported previously [10, 16]; however, the long-term trend of the participation rate by region and the analysis of this rate considering changes in the number of people per household has not yet been reported. Additionally, it is important to identify the proportion of the number of participants of the NHNS_J against the number of residents in the areas of the survey.

Therefore, in this study, the estimated participation rate (EPR) of the NHNS_J was defined considering the number of people per household, and its trends and annual percent change (APC) by region, gender, and age group were analyzed. This study aimed to identify EPR trends and its APC by region, gender, and age group in the NHNS_J from 1997 to 2019.

## Materials and methods

### Definition of estimated participation rate

**Fig 1:**
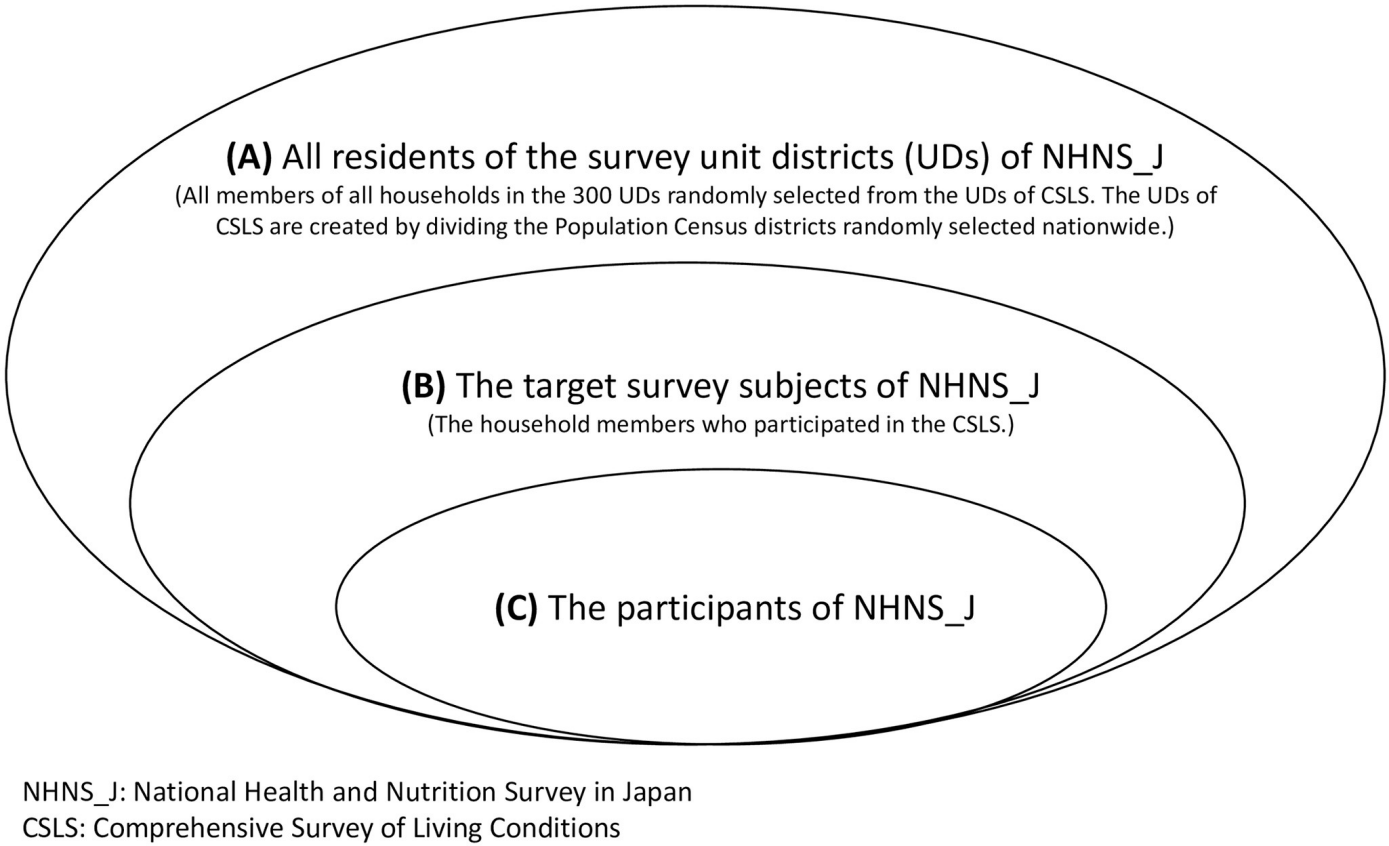
Survey districts and participants of NHNS_J. The estimated participation rate (EPR) is defined as C/A.

Fig 1 illustrates the survey districts of NHNS_J participants; 300 unit-districts (UDs) were randomly sampled from the larger number of UDs for the Comprehensive Survey of Living Conditions (CSLC) conducted before the NHNS_J in the same year (A), where UDs of CSLC were created by dividing the Population Census district geographically (approximately 5,500 Census districts for a large-scale CSLC every 3 years and approximately 1,100 Census districts for a small-scale CSLC in each interim year). In the sampled 300 UDs, NHNS_J subjects were household members aged ≥1 years, who also participated in the CSLC preceding the NHNS_J (B). NHNS_J (C) participants were excluded non-participants from (B).

Therefore, the participation rate of NHNS_J has been defined using the proportion of participants against the number of people who participated in the CSLC (= C/B) [4, 5].

However, as the participation rate of the CSLC is not 100%, NHNS_J subjects are not the same as all residents (all household members of all households = A) in NHNS_J UDs. Therefore, in this study, we defined EPR as the proportion of the number of NHNS_J participants against the number of residents in NHNS_J UDs (= C/A).

### Creating a database

This study was conducted in accordance with the Declaration of Helsinki. The NHNS_J data, which consisted of the Nutrition Intake Status Survey, Physical Status Questionnaire, and Lifestyle Habits Questionnaire, from 1997 to 2019, was obtained from the Ministry of Health, Labor, and Welfare (MHLW), Japan. The data was provided in electronic media through the prescribed MHLW procedures. The subjects in the database were anonymized, and there was no physical or mental intrusion on the subjects. Furthermore, the data were recorded after being encrypted in a specific computer under the information security policy of the researcher’s affiliated institution. The outside connection (including the hospital LAN) was cut-off during the analysis. The Ethics Committee of the National Institute of Public Health, Wako, Saitama, Japan agreed that there were no ethical issues with the above contents and approved the implementation of this study (NIPH-IBRA#12356, December 28, 2021).

We created a database according to the following steps:

**Step 1:** The data of those who responded to the Nutrition Intake Status Survey, Physical Status Questionnaire, or Lifestyle Habits Questionnaire were extracted from the NHNS_J database, and these participants were defined as “respondents.” The reason for using post-1997 data was that the Nutrition Intake Status Survey changed from the method for surveying household intake to surveying individual intake in 1995, which might have affected the survey burden in 1995–1996. After 1997, the survey method has been established. Next, a database was created to classify the number of participants by gender, prefecture, and age groups (age groups: 1–6, 7–14, 15–19, 20–29, 30–39, 40–49, 50–59, 60–69, 70–79, and ≥80 years), where the age groups were the same as those in the NHNS_J results reported by MHLW [1].
**Step 2:** From the published statistical e-Stat database, populations classified by prefecture, gender, and age group and the number of people per household by prefecture according to the 1997–2019 Population Census or population estimates [17] were obtained and added to the study database. The Population Census is a complete enumeration survey conducted every 5 years. The population by age group in the Population Census or population estimates is tabulated in ranges of 5 years, such as 0–4, 5–9, 10–14, and 15–19 years; however, the age groups for children in NHNS_J are 1–6 and 7–14 years. Therefore, to make the population data for the age groups of NHNS_J, the population aged 1–6 years was estimated to be four-fifth of the population of 0–4 years and two-fifth of the population of 5–9 years; the population aged 7–14 years was estimated in the same manner.
**Step 3:** Since the number of participants in some prefectures was too small to conduct statistical analysis, we analyzed the data by regional blocks. The prefectural data were recalculated as per the 12 regional blocks.

The MHLW defines a regional block as a unity of several divisions of national land and regions, and in NHNS_J, the whole country is classified into 12 regions [18].

The NHNS_J report shows the mean and standard deviation of the food group intakes by regional block [19]; the 12 blocks are displayed on the map (S1 Fig).

### Statistical analysis

Trends and APC analyses were performed by gender and regional block. The 2012 and 2016 NHNS_J surveys were excluded because the sampling method and number of subjects were different from those in other years [20, 21].

(1) The overall EPR of the NHNS_J by the age group for each year were calculated as follows:

- EPR = number of NHNS_J participants in 300 UDs/the number of residents in 300 UDs.
- Number of residents in 300 UDs ≈ the number of UDs (= 300) × mean number of residents per UD.
- Mean number of residents per UD ≈ mean number of households per UD × the mean number of people per household.
- The mean number of households per UD was estimated by equally dividing the number of households in each Census district, which was available from the e-Stat database [22], so that the number of households in each UD would be ≤30 according to the rule to create UDs [2, 23]. The mean number of people per household in each Census district was available from e-Stat and was assumed to be equal to that in each UD.
- Since the Population Census is a complete enumeration survey conducted every 5 years, the data in each interim year was estimated by the linear interpolation using data in 2000, 2005, 2010, 2015, and 2020 [24], e.g., the mean number of households per UD in 2001 was estimated by adding one-fifth of the difference in those between 2000 and 2005 to that in 2000.
- The number of people per household in each regional block was also obtained from the Population Census data (or linearly interpolated for each interim year).

The explanations above can be formulated as follows:

*y*: NHNS_J survey year (1997–2019, except 2012 and 2016),
*b*: regional block (1–12),
*a*: age group (10 groups),
*i*: index number of UD,
*P*_*y*,*b*,*a*_: population by Population Census or population estimates,
*n*_*y*,*b*,*a*,*i*_: number of participants in NHNS-J,
*N*_*y*,*b*,*a*,*i*_: number of all residents (all household members in all households),
*H*_*y*,*b*_: mean number of households per UD,
*M*_*y*,*b*,*a*_, *M*_*y*,*b*_: mean number of household members.
*M*_*y*,*b*_ was obtained from Population Census (or linear interpolation for other years).

By definition,

$${EPR}_{y,b,a}=\frac{\sum_{i}n_{y,b,a,i}}{\sum_{i}N_{y,b,a,i}},$$

where,

$$N_{y,b,a,i}\approx H_{y,b}\times M_{y,b,a}.$$

The age-group-specific *M*_*y*,*b*,*a*_ was estimated in proportion to the age distribution of the population of the regional block as

$$M_{y,b,a}\approx M_{y,b}\times \frac{P_{y,b,a}}{\sum_{a}P_{y,b,a}}.$$

(2) The trends of the EPR overall and by age group were graphically shown with an exponential regression curve regressed on the survey year.

(3)APC, or relative change per year, was calculated as $\left(e^{b_{1}}-1\right)\;\times \;100\;\left(\%\right)$, where *b*_1_ is the regression coefficient for 1 year by the exponential regression. The standard error and statistical significance of EPR by age group were also calculated. Additionally, the mean EPR for 1997–1999 (first 3 years of this study) was added to the graph as supplementary material to illustrate the relationship between initial EPR and subsequent APC by age group. Finally, the Joinpoint regression analysis was performed to examine whether there was a trend change point (joinpoint) or not [25]. If a joinpoint is suggested, the average APC (AAPC), which is a weighted average of APCs before and after the joinpoint, is also calculated.

## Results

The number of households per UD was almost constant because of the rule to create UDs and ranged 23.3–24.0 in 2000–2020 (Table 1), while the number of people per household decreased in all regional blocks (by 17% nationwide). As a result, the total number of people living in 300 UDs for NHNS_J should have decreased.

**Table 1:** Nationwide and regional block-wise number of households per unit-district and members per household in Japan.

Regional block	Nationwide				Hokkaido				Tohoku				Kanto I				Kanto II			
	Households per UD		Members per household		Households per UD		Members per household		Households per UD		Members per household		Households per UD		Members per household		Households per UD		Members per household	
year	Mean	SD	Mean	SD	Mean	SD	Mean	SD	Mean	SD	Mean	SD	Mean	SD	Mean	SD	Mean	SD	Mean	SD
2000	23.5	3.5	2.7	0.9	23.6	3.5	2.4	0.8	23.6	3.5	3.0	1.0	23.5	3.4	2.5	0.7	23.6	3.5	3.0	0.8
2005	23.5	3.5	2.6	0.9	23.5	3.6	2.3	0.9	23.6	3.5	2.9	1.0	23.6	3.4	2.4	0.9	23.6	3.5	2.8	0.8
2010	23.6	3.5	2.4	0.8	23.5	3.6	2.2	0.7	23.6	3.5	2.7	0.9	23.7	3.4	2.3	0.8	23.8	3.5	2.7	0.8
2015	23.7	3.5	2.4	0.9	23.6	3.5	2.1	0.7	23.6	3.7	2.6	0.9	23.8	3.4	2.2	0.9	23.8	3.4	2.6	0.7
2020	23.8	3.4	2.2	0.9	23.6	3.5	2.1	1.0	23.7	3.6	2.4	0.8	24.0	3.3	2.1	1.0	23.9	3.4	2.4	0.6
Regional block	Hokuriku				Tokai				Kinki I				Kinki II				Chugoku			
	Households per UD		Members per household		Households per UD		Members per household		Households per UD		Members per household		Households per UD		Members per household		Households per UD		Members per household	
year	Mean	SD	Mean	SD	Mean	SD	Mean	SD	Mean	SD	Mean	SD	Mean	SD	Mean	SD	Mean	SD	Mean	SD
2000	23.5	3.5	3.1	0.9	23.5	3.5	2.9	1.3	23.4	3.6	2.6	0.7	23.3	3.7	2.9	0.8	23.5	3.6	2.7	0.8
2005	23.5	3.6	2.9	0.9	23.5	3.5	2.7	1.1	23.5	3.6	2.5	0.9	23.3	3.7	2.8	0.6	23.5	3.6	2.6	1.0
2010	23.6	3.6	2.8	0.8	23.6	3.5	2.6	1.1	23.6	3.6	2.4	0.8	23.5	3.7	2.6	0.7	23.5	3.6	2.5	0.7
2015	23.6	3.5	2.7	1.0	23.7	3.5	2.5	0.9	23.6	3.6	2.3	0.9	23.5	3.7	2.5	1.2	23.6	3.6	2.4	0.7
2020	23.7	3.5	2.5	0.7	23.8	3.4	2.4	0.9	23.7	3.5	2.2	1.0	23.6	3.7	2.4	1.1	23.6	3.6	2.3	0.7
Regional block	Shikoku				Kita Kyushu				Minami Kyushu											
	Households per UD		Members per household		Households per UD		Members per household		Households per UD		Members per household									
year	Mean	SD	Mean	SD	Mean	SD	Mean	SD	Mean	SD	Mean	SD								
2000	23.7	3.5	2.7	0.8	23.5	3.5	2.7	0.9	23.7	3.4	2.7	0.8								
2005	23.6	3.6	2.5	0.8	23.5	3.6	2.6	0.9	23.7	3.4	2.6	0.8								
2010	23.7	3.5	2.4	0.7	23.6	3.6	2.4	0.9	23.8	3.4	2.5	0.7								
2015	23.8	3.5	2.3	0.6	23.6	3.6	2.4	1.0	23.8	3.5	2.4	0.6								
2020	23.7	3.5	2.2	0.5	23.6	3.5	2.2	0.8	23.8	3.5	2.3	0.6								

Data: Population Census in 2000, 2005, 2010, 2015, and 2020, UD: unit-districts, SD: standard deviation

**Fig 2:**
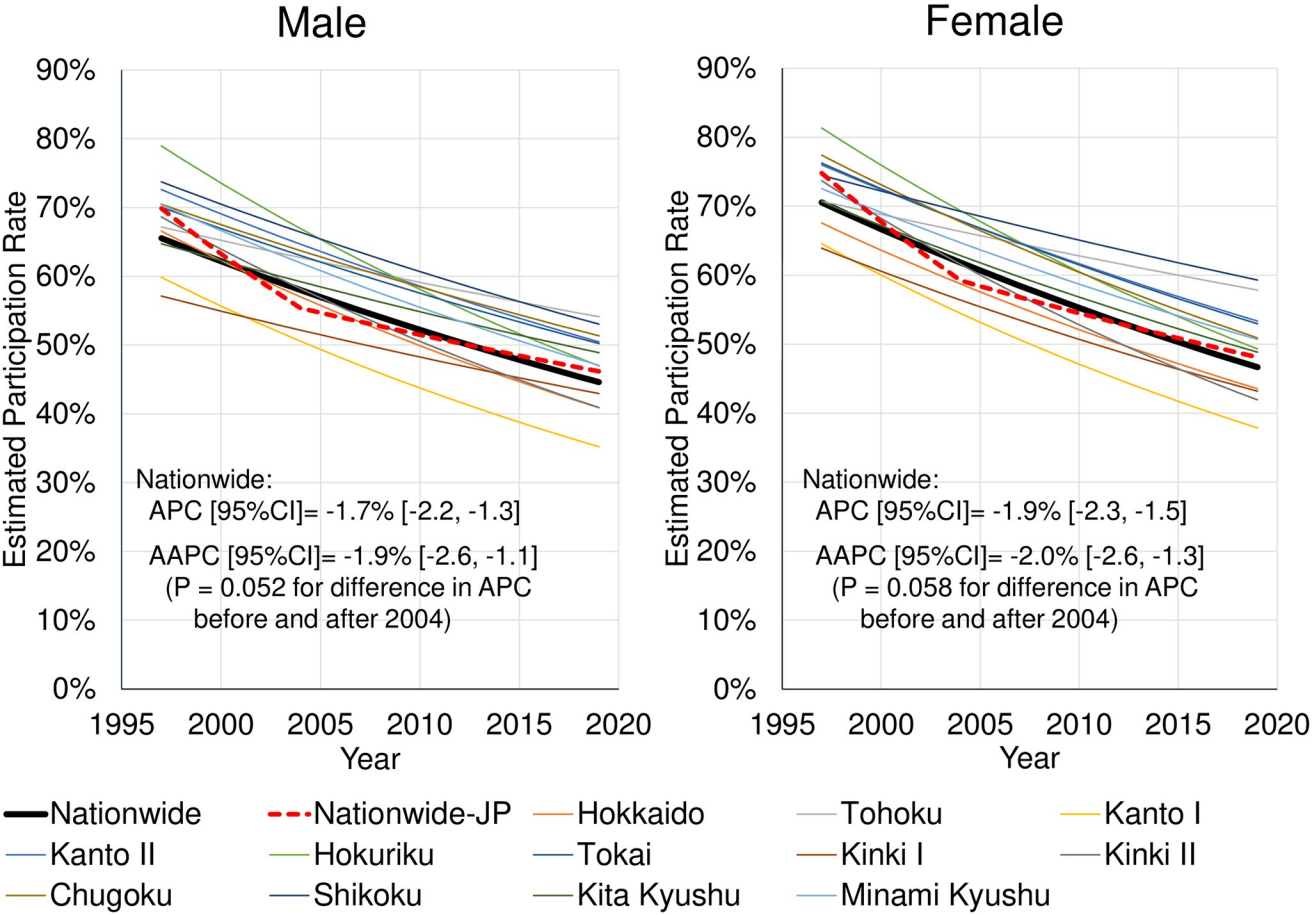
Summary of the secular trends in estimated participation rates (EPR) nationwide and by regional block. APC: annual percent change. AAPC (average APC): a weighted average of the APCs before and after 2004. Nationwide-JP: nationwide Joinpoint regression curve with one joinpoint. See Fig 3 for details.

**Fig 3:**
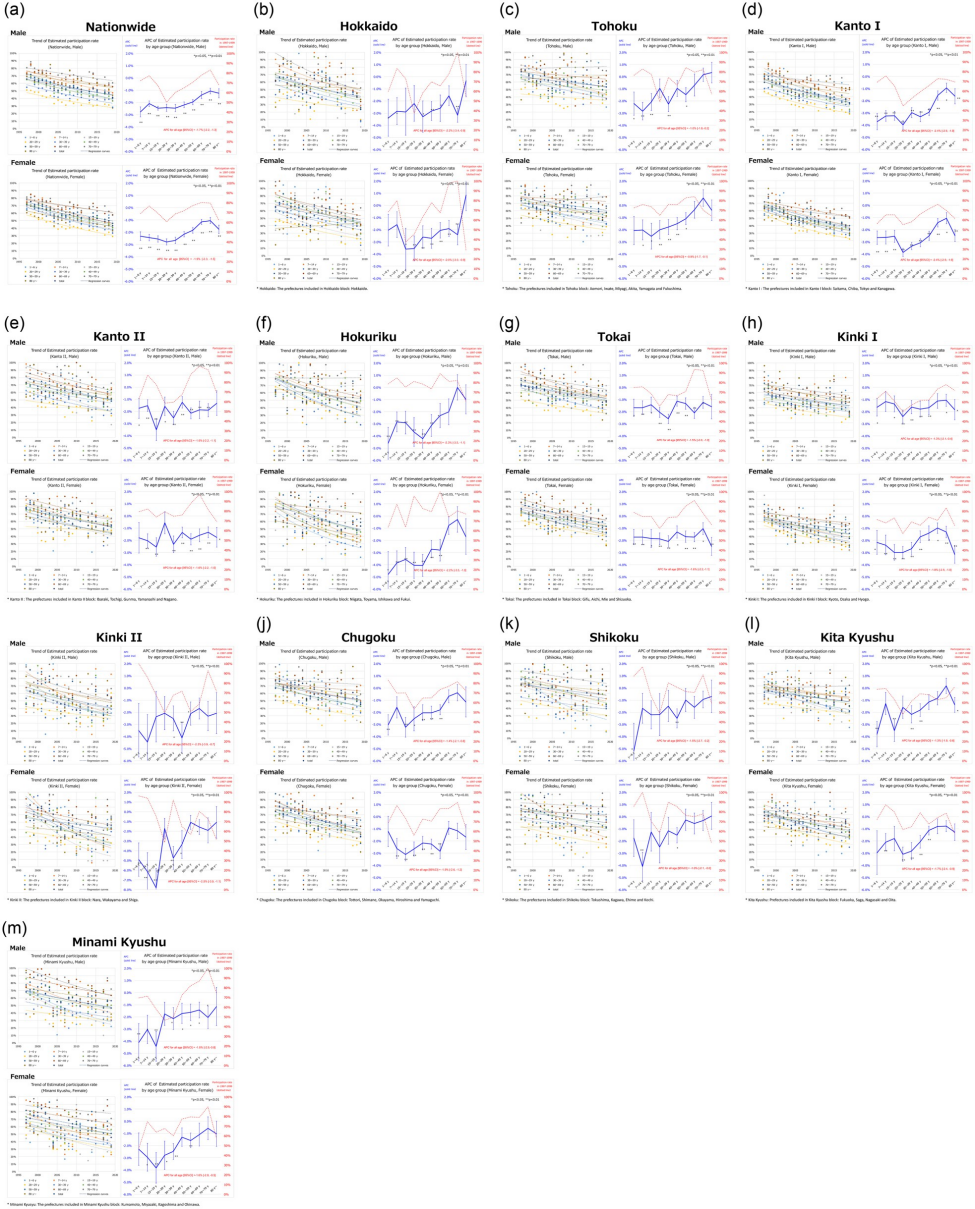
Secular Trend of Estimated Participation Rate (EPR) (left) and Annual Percent Change (APC) (right) nationwide and by regional block, gender and age group. Regional blocks: Hokkaido, Tohoku, Kanto I, Kanto II, Hokuriku, Tokai, Kinki I, Kinki II, Chugoku, Shikoku, Kita Kyushu, Minami Kyushu. See S1 Fig for geographic location.

**Fig 4:**
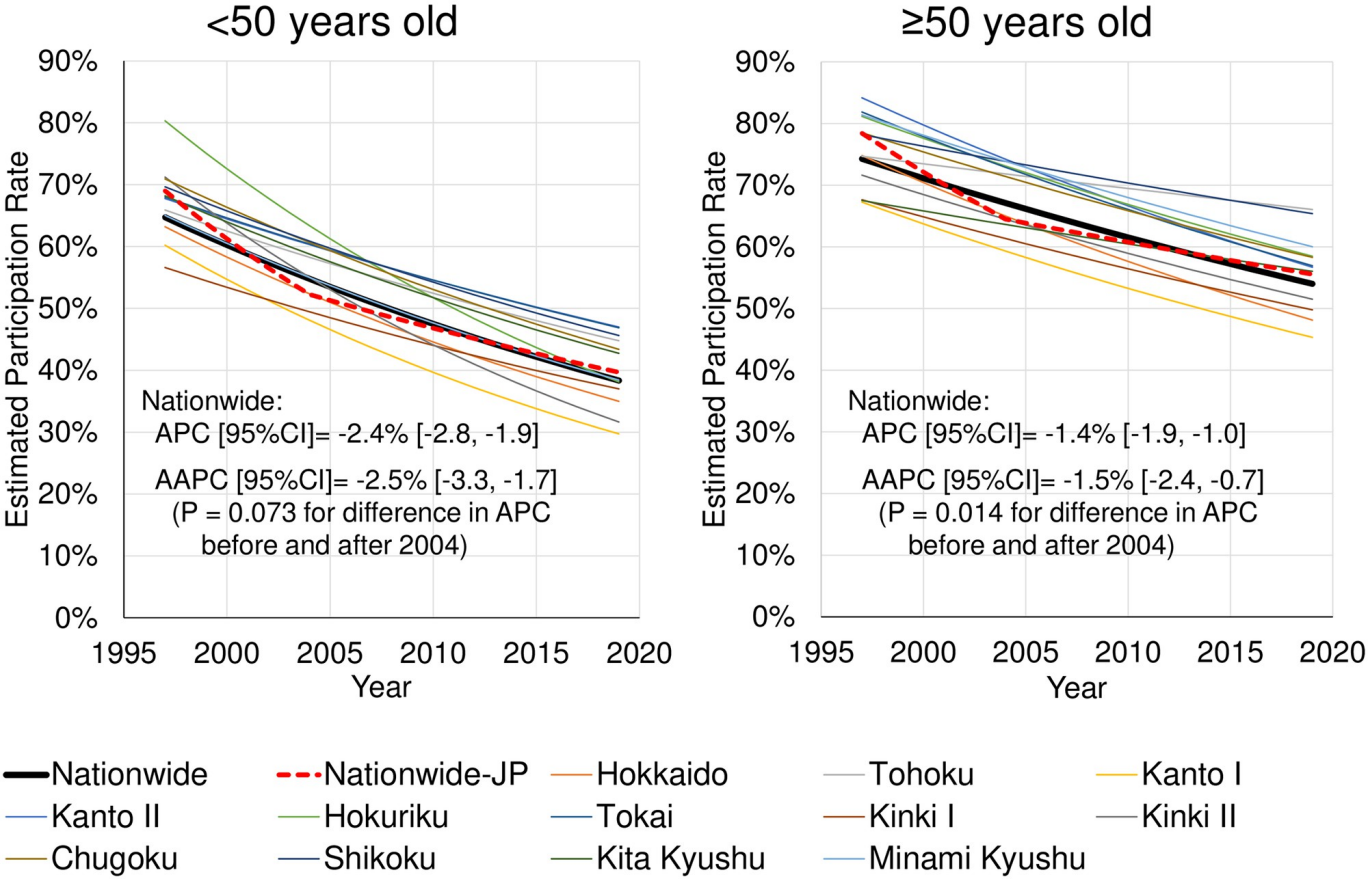
Comparison of trends in estimated participation rates for age groups <50 years and ≥50 years for both sexes. P = 0.001 for difference in annual percent change (APC) between the age groups. AAPC (average APC): a weighted average of the APCs before and after 2004. Nationwide-JP: nationwide Joinpoint regression curve with one joinpoint.

The secular trends of EPR nationwide and by regional block are summarized in Fig 2 as the exponential regression curves. The nationwide APC [95% confidence interval (CI)] was −1.7% [−2.2, −1.3] for males and −1.9% [−2.3, −1.5] for females. The EPR in all regional blocks decreased (APC ranged -2.4% to -1.0% for males and -2.5% to -1.0% for females, values not shown in graph). In other words, differences in APC decline by regional block were noted. The detailed graphs are also shown in Fig 3. The graph (left) shows the secular trends (exponential regression curve) of EPR by the age group. For male and female, the EPR had been declining nationwide and in all regional blocks in most age groups. In particular, there was a large decline in all age groups in Kanto I, Kanto II, Tokai, and Kinki I blocks. The graph (right) shows APC (thick solid line), standard error (vertical line), and statistical significance (*p < 0.05, **p < 0.01) of EPR by the age group. In Kanto II, Tokai, and Kinki I blocks, no differences were noted among age groups; however, in other regional blocks, the decline in younger age groups (particularly 20s, 30s, and 40s) was larger than in older age groups. The decline in APC nationwide in the younger age groups (especially in those in their 20s and 30s) was larger than that in the older age groups. The nationwide APC in the age group <50 years was significantly larger than in the age group ≥50 years (APC [95% CI]: -2.4% [-2.8, -1.9] and -1.4% [-1.9, -1.0], respectively, p = 0.001 for difference in APC) (Fig 4).

**Fig 5:**
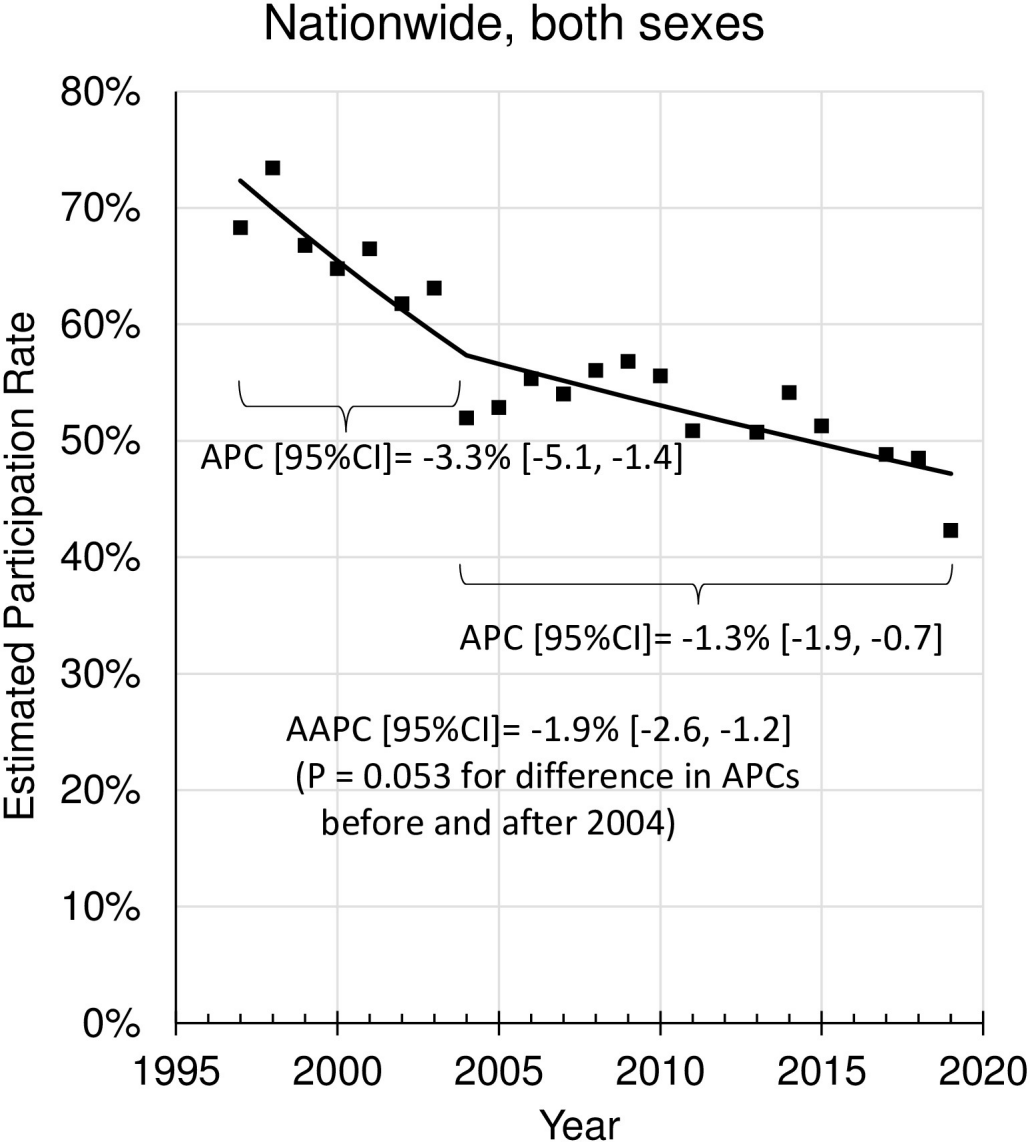
Change in the nationwide annual percent change (APC) before and after 2004. AAPC (average APC): a weighted average of the APCs before and after 2004. P = 0.053 for difference in APC before and after 2004 by the Joinpoint regression analysis.

Furthermore, the Joinpoint regression analysis showed that the EPR declined rapidly during 1997 to 2003 (APC = -3.3%), followed by a sudden drop in 2004, and a smaller APC after 2004 (APC = -1.3%) although the change in APC before and after 2004 was not significant at a marginal level (p = 0.053) (Fig 5). Similarly, non-significant changes in APC before and after 2004 were also seen in males and females (Fig 2) and in the age group <50 years and in the age group ≥50 years (Fig 4).

## Discussion

In this study, we clarified EPR trends by regional block, gender, and age group in the NHNS_J from 1997 to 2019. In Health Japan 21 (the second term), important indicators, including nutritional status (e.g., body mass index), salt intake, and vegetable intake, of nutrition and dietary habits were evaluated using the NHNS_J data [3]. As the goal is to reduce health inequalities, it would be extremely important to assess the trends of the participation rate in NHNS_J by regional blocks.

This study newly confirmed that EPRs had declined in all regional blocks, and APC decline in younger age groups, mainly those in their 20s–40s, was larger than in older age groups in many regional blocks. In particular, it was a novel finding from this study that there was a significant difference in the rate of decline in participation rates between under 50 and over 50 years old groups.

Changing lifestyle of the young generation in Japan is one of the reasons for the declining trend in participation rates, with reports showing an increase in the number of people living alone and in apartments instead of houses [26, 27]. In addition, when many non-participants in the survey have a low income and are from younger generations, they may feel they have no monetary incentives to participate in surveys. Other reasons include concerns about privacy and confidentiality, decreased interest in social contribution, and a general decline in volunteerism [11].

Furthermore, it was new finding that the EPR nationwide dropped significantly in 2004 and the APC changed smaller thereafter. Japan’s total population peaked in 2008 and has been declining ever since [28]. In addition, the Niigata Prefecture Chuetsu Earthquake (seismic intensity 7, 3,175 houses were completely destroyed, 13,810 houses were partially destroyed, etc.) on October 23, 2004 [29]. It might have had a big impact on the participation rate, because the NHNS_J was implemented in November and many local government officials were dispatched to Niigata from other prefectures to support the reconstruction efforts. It cannot be denied that the trend of EPR and APC may be affected by factors such as Japan’s population decline and changes in people’s lives due to disasters. This analysis will be the issue of future research.

Several reports exist also on the participation rates of national surveys in other countries [30, 31]. It has been shown that the younger age groups’ participation rate is low in Finland [32]. In Denmark, ethnic minorities, place of residence, language, and presence of illness affected the decline in participation rates [33, 34]. In some countries, an association between participation rates and socioeconomic status or gender has been reported [13, 30, 35].

Several studies had shown that the low participation rate was related to potential factors including area, location, target group (age and gender), survey topic, survey delivery method, and question type (single or multiple choice) [15, 36, 37]. However, the reports did not show the trends of regional differences for long periods. This study can be a meaningful medium to identify them.

Historically, NHNS has been conducted through home visits and face-to-face surveys at specific venues. However, in recent years, MHLW has initiated accepting survey forms returned by multiple survey methods, including mail or electronic surveys, since it may be effective to have the subjects select a response method from a range of different formats to increase the participation rate. In addition, some survey areas have tried handing out the reward to participants before conducting the survey [11]. It will be necessary to identify the effects of these methods on the participation rate of the younger generation in the future.

Additionally, since it has been reported that there are regional differences in the age–class structure of populations in Japan [22, 24], this study corrected the population of age groups and showed the participation rate trends by regional block.

From 1945 until the present, NHNS_J has surveyed almost the same number of UDs (300 UDs) and households. Although the number of people per household has decreased [12], as this study also identified, the number of households in UDs has not been considered for the sampling design of NHNS_J. Thus, the target survey subjects of the NHNS_J have decreased because the mean members per household have decreased from 2.7 in 2000 to 2.2 in 2020. In the future, it is expected that the number of people per household and the composition of members of households will change, along with the changes in demographic structure due to Japan’s declining birthrate and aging population [28, 38]. When predicting the NHNS_J participation rate, it may be necessary to perform an analysis that considers the number of people per household. In addition, it will be necessary to consider whether the district sampling method is acceptable, and what can be done to increase the target population of the young generation.

Conversely, the households headed by foreigners were only 1.2% in 2000 and 2.4% in 2020 [17] and were excluded from NHNS_J [39]. However, the number of foreigners is projected to grow rapidly in Japan in the coming decades [40]. The participation of non-applicable persons including foreigners in Japan may be considered for NHNS sampling design in the future, although it did not focus on non-applicable people in this study.

This study has some limitations. The mean number of households per UD was used instead of the actual number in each randomly sampled UD. This may cause a random error in estimating the number of all residents in UDs. Since the households headed by foreigners are excluded from NHNS_J, the increase in their proportion (1.2% in 2000 and 2.4% in 2020) may have a very minor impact on the decreasing EPR trend in this study. Additionally, the database of this study was based on the data of NHNS_J respondents. As the situation of the non-respondents was unknown, it may be necessary to link it with the CSLC data, which is the population of the survey, to clarify the characteristics of non-respondents. Further studies should consider this issue.

## Conclusion

This study aimed to identify the trend of approximate participation rate in NHNS_J. EPR was estimated by regional block, gender, and age group, and the trend and APC were clarified. In the results, trends of EPRs in all regional blocks declined, and the decline in nationwide APC in the younger age group <50 years was larger than that in the age group ≥50 years.

## Supporting information

S1 Fig Map of regional blocks and prefectures in Japan. (PPTX)

S1 Table The data set underlying the results. (XLSX)
