# Supplementary material for: Trend of estimated participation rate by regional block, gender, and age group in the 1997–2019: National Health and Nutrition Survey in Japan
Source: PLoS One. 2024 Mar 13;19(3):e0286169. doi: 10.1371/journal.pone.0286169 (PMC10936830; doi:10.1371/journal.pone.0286169)
Supplement: S1 Fig — (PPTX) [file pone.0286169.s001.pptx]

## Slide 1
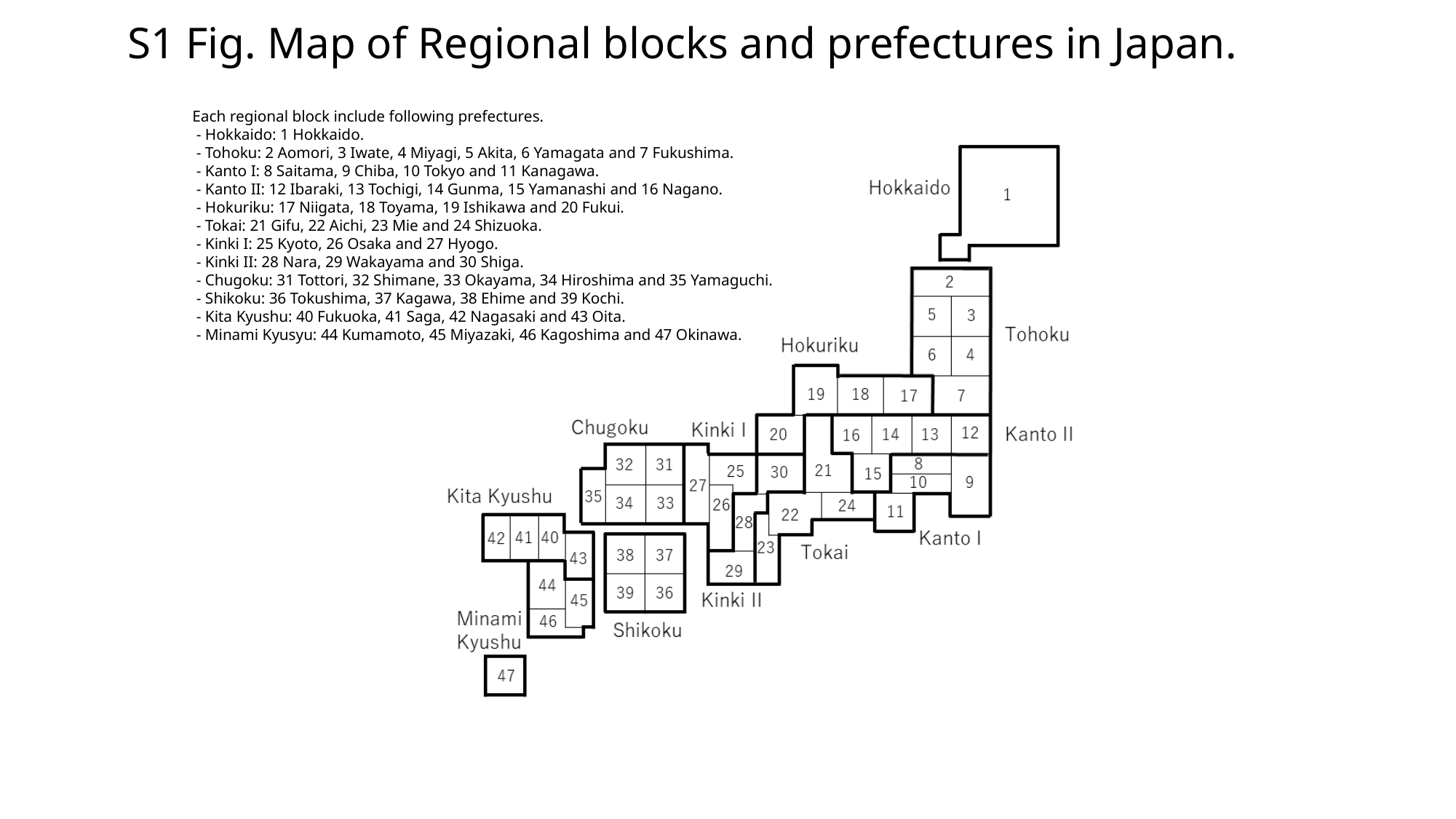

S1 Fig. Map of Regional blocks and prefectures in Japan.
Each regional block include following prefectures.
 - Hokkaido: 1 Hokkaido.
 - Tohoku: 2 Aomori, 3 Iwate, 4 Miyagi, 5 Akita, 6 Yamagata and 7 Fukushima.
 - Kanto I: 8 Saitama, 9 Chiba, 10 Tokyo and 11 Kanagawa.
 - Kanto II: 12 Ibaraki, 13 Tochigi, 14 Gunma, 15 Yamanashi and 16 Nagano.
 - Hokuriku: 17 Niigata, 18 Toyama, 19 Ishikawa and 20 Fukui.
 - Tokai: 21 Gifu, 22 Aichi, 23 Mie and 24 Shizuoka.
 - Kinki I: 25 Kyoto, 26 Osaka and 27 Hyogo.
 - Kinki II: 28 Nara, 29 Wakayama and 30 Shiga.
 - Chugoku: 31 Tottori, 32 Shimane, 33 Okayama, 34 Hiroshima and 35 Yamaguchi.
 - Shikoku: 36 Tokushima, 37 Kagawa, 38 Ehime and 39 Kochi.
 - Kita Kyushu: 40 Fukuoka, 41 Saga, 42 Nagasaki and 43 Oita.
 - Minami Kyusyu: 44 Kumamoto, 45 Miyazaki, 46 Kagoshima and 47 Okinawa.
